# Usefulness of cultivar-level calibration of AquaCrop for vegetables depends on the crop and data availability

**DOI:** 10.3389/fpls.2023.1094677

**Published:** 2023-03-09

**Authors:** Willem Coudron, Pieter De Frenne, Kris Verheyen, Anne Gobin, Charlotte Boeckaert, Tim De Cuypere, Peter Lootens, Sabien Pollet, Tom De Swaef

**Affiliations:** ^1^Plant Science Unit, Research Institute for Agriculture, Fisheries and Food (ILVO), Melle, Belgium; ^2^Forest & Nature Lab, Department of Environment, Ghent University, Gontrode, Belgium; ^3^Remote Sensing, Flemish Institute for Technological Research (VITO), Mol, Belgium; ^4^Department of Earth and Environmental Sciences, Faculty of Bioscience Engineering, KU Leuven, Leuven, Belgium; ^5^Vlaams Kenniscentrum Water (VLAKWA), Flemish Institute for Technological Research (VITO), Kortrijk, Belgium; ^6^Department of Outdoor Horticulture And Precision Agriculture, Inagro, Rumbeke-Beitem, Belgium

**Keywords:** Bayesian calibration, crop model, decision support tool, spinach, cauliflower, sensitivity analyis

## Abstract

As a result of climate change, climatic extremes are expected to increase. For high-value crops like vegetables, irrigation is a potentially economically viable adaptation measure in western Europe. To optimally schedule irrigation, decision support systems based on crop models like AquaCrop are increasingly used by farmers. High value vegetable crops like cauliflower or spinach are grown in two distinct growth cycles per year and, additionally, have a high turnover rate of new varieties. To successfully deploy the AquaCrop model in a decision support system, it requires a robust calibration. However, it is not known whether parameters can be conserved over both growth periods, nor whether a cultivar dependent model calibration is always required. Furthermore, when data are collected from farmers’ fields, there are constraints in data availability and uncertainty. We collected data from commercial cauliflower and spinach fields in Belgium in 2019, 2020 and 2021 during different growing periods and of different cultivars. With the use of a Bayesian calibration, we confirmed the need for a condition or cultivar specific calibration for cauliflower, while for spinach, splitting the data per cultivar or pooling the data together did not improve uncertainty on the model simulations. However, due to uncertainties arising from field specific soil and weather conditions, or measurement errors from calibration data, real time field specific adjustments are advised to simulations when using AquaCrop as decision support tool. Remotely sensed or *in situ* ground data may be invaluable information to reduce uncertainty on model simulations.

## 1 Introduction

Because of increased frequencies of extreme events such as heat waves and droughts, it is increasingly important to adapt our agricultural practices to these changing conditions (Challinor et al., 2014). For high-value vegetable crops, sustainable use of irrigation can be part of an integrated strategy of adaptation to drier conditions, next to improvement of soil structure and quality, breeding for adapted varieties, growing alternative species or use of mixed cropping systems such as agroforestry. Sustainable use of irrigation should prevent depletion of water sources and safeguard agricultural soil quality. Therefore, we need to optimize the timing and amount of irrigation water. Crop models like AquaCrop have been used extensively as part of a decision support tool for irrigation water management (e.g. Cusicanqui et al., 2013), to characterize the response of crops to water stress (Geerts et al., 2010; Abedinpour et al., 2012), to quantify crop water use (Gobin et al., 2017; Kersebaum et al., 2016), and to assess the impact of climate change on crop production (Vanuytrecht et al., 2014). This model has been developed by the land and water division of the Food and Agriculture Organization of the United Nations (FAO) and is characterized by its simplicity and robustness. It provides insight into the development of crops in response to environmental conditions and is especially well suited for conditions where water is the limiting factor (Hsiao et al., 2009; Raes et al., 2009; Steduto et al., 2009; Vanuytrecht et al., 2014).

Before crop models can be used as part of a decision support system to guide management practices, it is of great importance that parameters are properly calibrated. However, model calibration requires a substantial amount of data (Coudron et al., 2021). Ideally, the model calibration can be done per crop species and is valid over a wide range of plausible conditions. This should ensure robust model performance and adequate crop growth predictions for the targeted population of environments (cf. mega-environment) and the crop species. However, as a result of climate change, spatial and temporal variation in climate is expected to increase (van Oldenborgh et al., 2013). Second, also the turnover rate of new varieties for vegetable crops is high. As a result, it is not well-known how robust the AquaCrop model performs over this extended range of conditions, and whether a cultivar dependent model calibration is always required.

Additionally, when using AquaCrop as a decision support tool for generic advice about field management, data sources will often be used with different levels of uncertainty ranging from soil maps, weather data and management conditions which may or may not be provided by government instances or farmers. As such, uncertainties regarding input and output for model simulations are a given.

Here we aim to evaluate the stability of AquaCrop parameters across environmental conditions and cultivars for two (often irrigated) vegetable crops in Flanders, spinach (*Spinacia oleracea* L., Amaranthaceae) and cauliflower (*Brassica oleracea* L., Brassicaceae). For both of these crops we collected field data across different locations and for different cultivars. Both crops are cultivated twice per growing season (early: April-July and late: July-October) and thus provide data in substantially different weather conditions.

We use a Bayesian calibration framework to calibrate the AquaCrop model, which allows us to assess prediction uncertainty. This is considered to be a standard part of modelling to determine liability of model simulations (Wallach and Thorburn, 2017). Thereby we altered the stratification of the available data into different groups: all available data, data per cultivar and data per growing period. The resulting parameter distributions are used to simulate the uncertainty in model outputs which are compared in terms of mean values and mean absolute deviation (MAD). Finally, the accuracy and uncertainty of the model on these levels is assessed to highlight the potential need for model adjustment as a decision support tool.

## 2 Materials and methods

### 2.1 AquaCrop: Model description

AquaCrop is a water-driven production model which is able to simulate plant growth of several herbaceous crops. As such, it is well suited for conditions in which water is the main limiting growth factor and thus makes it an interesting model for irrigation advice in drought sensitive regions. At each simulation time step, yield is the ultimate model output, resulting from a number of calculation steps. First, the green canopy cover is calculated based on the available soil water content. This canopy cover allows to separate crop transpiration and soil evaporation and thus avoids the confounding effect of the nonproductive consumptive use of water. The amount of water transpired by the crop is then linked to the amount of biomass produced through the water productivity (WP), which is one of the crucial parameters linking transpiration to biomass production. Lastly, a specified fraction of the biomass is apportioned to the final yield by the harvest index parameter. During these calculation steps, different stress coefficients are introduced for water, heat, cold and soil fertility. Most importantly, four water stress thresholds define how canopy development is limited. As such there is a threshold for: (1) canopy expansion, (2) stomatal closure, (3) early canopy senescence, and (4) aeration stress (as a result of an excess of water in the soil). AquaCrop is a generic model and thus can simulate growth of a variety of crop species. Therefore, the model should be parameterized by altering conservative and non-conservative parameters. Conservative parameters are typically fixed for a crop species, while non-conservative may vary with crop cultivar. AquaCrop can be freely downloaded at (http://www.fao.org/nr/water/aquacrop.html). More information on the model is provided by Steduto et al. (2009) and Raes et al. (2009).

### 2.2 Study region

**Figure 1:**
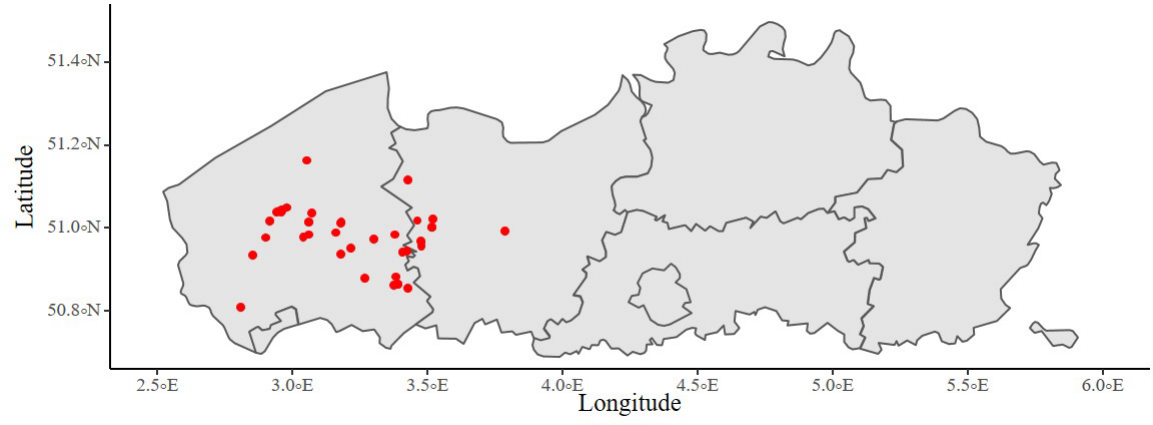
All field locations monitored of cauliflower and spinach in Flanders during the growing seasons of 2019, 2020 and 2021.

To collect the required calibration and validation data, actual fields used by commercial farmers were monitored in Belgium during the years 2019, 2020 and 2021 thereby capturing a variety of weather, soil conditions and crop cultivars (**Table S1** and **Figure 1**). In total, sixteen cauliflower fields were monitored from cultivars Giewont (Seminis) and David (Syngenta), and twelve spinach fields from cultivars Berkner (Seminis), Bonobo (Rijk Zwaan), Eagle (Rijk Zwaan), Puma (Rijk Zwaan), Sacramento (Pop Vriend), Spirico (Nunhems) and Whale (Rijk Zwaan). Soil texture classes in these fields were determined according to the Belgian soil classification system as sand, loamy sand, sandy loam and loam. Additionally, farmers were requested to inform us about irrigation timing and amounts and to communicate about other management practices. For multiple fields, soil EC (electric conductivity), pH and organic matter were determined at the start of the growing season.

### 2.3 Crop and microclimate data collection

Some of the most important output variables for AquaCrop calibration are yield, biomass, canopy cover and soil moisture content (Coudron et al., 2021). Therefore, these variables were monitored during the growing periods in 2019, 2020 and 2021 on actual commercial fields (**Table S1**). To use AquaCrop as a decision support tool in Flanders, weekly to bi-weekly data were gathered in different environmental conditions for calibration on a total of 28 fields (sixteen of cauliflower and twelve of spinach).

#### 2.3.1 Sampling biomass and yield

Destructive samples of the cauliflower plants were taken approximately every two weeks from the time of planting to the time of final harvest. Spinach was sampled more frequently (approximately every week) to guarantee sufficient data because of its short growing period. Yield for cauliflower was determined as the dry weight of the heads, while biomass was determined as the total aboveground dry weight of the entire plant.

For spinach, plants were sampled on a surface area of 40 by 40 cm at six locations in the field and the number of plants were counted to determine the sowing density. For cauliflower, four plants were harvested at two locations in the field. Afterwards, samples of biomass and yield were dried for 72 h at 70°C and weighed to determine the dry matter yield and dry matter biomass. Based on the plant or sowing density, dry matter values could afterwards be converted to ton ha^-1^ as the corresponding unit used by AquaCrop.

#### 2.3.2 Determining the canopy cover

To determine canopy cover, pictures were taken above the crops (at 1.5 m above the soil surface) with a mobile camera (Samsung galaxy j6 and Samsung galaxy a6) at fifteen randomly selected locations in the field at the same places soil samples were taken for determining soil moisture contents. Afterwards, these pictures were processed with ImageJ (https://imagej.nih.gov/ij/) to determine the percentage ground cover of the crop.

#### 2.3.3 Soil water content

Manual soil samples were taken every two weeks with an auger at 0-30cm and 30-60cm depth for cauliflower fields at fifteen locations in the field and were pooled for analysis. For spinach fields, samples were only taken in the 0-30 cm soil layer because roots of spinach do not grow in deeper soil layers. Soil samples were dried in the oven at 105°C and weighed after one day to obtain gravimetric water content. Bulk density was determined using Kopecky rings. With this information, the volumetric water content of the soils was calculated.

Additionally, on every field, 10HS soil moisture sensors (Geobas Pro, Vantage Agrometius) were installed for monitoring volumetric moisture content at two different depths, at 15 cm and 45 cm in cauliflower fields and at 10 cm and 35 cm in spinach fields. Additionally, the Geobas station also monitored soil temperature, precipitation and soil water retention at two depths.

#### 2.3.4 Meteorological data

AquaCrop needs a number of meteorological data as input variables, such as daily precipitation, maximum daily temperature, minimum daily temperature and reference daily evapotranspiration as calculated *via* the Penman-Monteith equation (Howell and Evett, 2004). Precipitation was measured locally at every field by the Geobas stations. Other meteorological data, or missing precipitation data, were obtained from the Royal Meteorological Institute of Belgium (RMI) which provides daily interpolated climatic data on a resolution of 5 by 5 km in Flanders.

### 2.4 Calibration procedure

#### 2.4.1 Sensitivity analysis

Before starting the Bayesian calibration, we performed the Morris method (Morris, 1991) as modified by Campolongo et al. (2007) from the sensitivity package (Iooss et al., 2020) in R [R version 4.1.2, RStudio Team (2020)] to determine the most important parameters in the studied environmental conditions. The Morris method is a global screening method which randomly samples the parameter space and changes every parameter one at the time (OAT) after which the mean of the absolute values of the elementary effects for all parameters (µ*) are calculated. However, for screening the effect of parameters on all the output variables, the elementary effect vectors were rescaled with the mean output of the simulations for the specific output variable (Campolongo et al., 2007). To get a general idea of the sensitivity of all output variables over the growing season, we averaged the sensitivity functions over the whole growing season and over all the output variables measured in the calibration fields. This way, non-influential or low influential parameters could be identified and removed from the Bayesian calibration. These parameters were fixed to set values based on literature and expert knowledge. The Morris method was performed with the parameter space divided in eight levels and the number of replicates was chosen to be 100 (Vanuytrecht et al., 2014; Coudron et al., 2021).

#### 2.4.2 Bayesian calibration

Bayesian analysis allows to get insight on the uncertainty of parameter estimations, and the subsequent model output. With our approach, we aim to dissect whether the AquaCrop model needs to be calibrated at the cultivar level for two vegetable types: a leafy vegetable (spinach) and a cabbage crop (cauliflower). Both crops are most commonly grown in two distinct growth periods and have a rapid turnover in terms of varieties. To ensure robust decision support systems based on crop models such as AquaCrop, model parameters should preferably be transferable across years, growth periods and cultivars.

The basic idea behind a Bayesian approach is to increase the belief concerning the probability of an event through the incorporation of observational data. Bayesian calibration is used to generate likelihood distributions instead of optimizing the model to a specific point in parameter space which is commonly performed in many calibration methods (Price, 1977; Powell, 2009). This framework starts with defining the uncertainty or prior distribution of the parameter values after which the prior belief is updated with observational data through application of Bayes’ theorem: P(ϑ|D) = P(ϑ) x P(D|ϑ)/P(D), where P(ϑ) represents the prior distribution and P(ϑ|D) the posterior distribution for the parameter ϑ given the input data D, P(D|ϑ) is the likelihood of the data given model output using parameters ϑ, and P(D) is a normalization constant. The posterior distribution allows to quantify the predicted uncertainty on the parameter values. This may be used to predict the subsequent uncertainty in model output by sampling parameter values from the posterior distribution and run model simulations. In this study, we used the ModMCMC function from the FME package (Soetaert and Petzoldt, 2010) in R (R version 4.1.2). This function performs a (MCMC) Markov Chain Monte Carlo simulation using an adaptive metropolis algorithm including a delayed rejection procedure which is the optimization algorithm used to determine whether to reject or accept the proposed jump. Gao et al. (2020) explain that the use of MCMC, coupled with the estimation of model error variance is a promising calibration method for the quantification of prediction uncertainty.

The number of iterations used to perform the Bayesian calibration was 30,000, with a burn-in-length of 3,000 and a jump of one tenth of the range between every parameter. Distributions of the parameters were set to be uniform, making sampling between the parameter ranges equally likely. The calibration was performed with the collected time series data from yield, biomass, canopy cover and water content (**Table S1**) pooled over the studied levels (See section 2.4.4 Data stratification).

#### 2.4.3 Calibration and sensitivity settings

In both the sensitivity analysis and the Bayesian calibration method, it is important to give plausible parameter ranges. **Table S2** shows the parameter ranges used for spinach and cauliflower, these ranges were determined based on literature values and expert knowledge. Since spinach is a short growing crop, several parameters that only become relevant in a later stage of the crop development, were not considered in this study. Additionally, when performing Bayesian calibration, explicit assumptions have to be made about the distribution of errors, whether the errors have the same distribution and whether errors for different variables are correlated (Wallach et al., 2021). In this study, we assumed the errors for all output variables to have the same distribution and that they are not correlated. These assumptions were tested using a Levene test using the R function leveneTest in the package ‘car’ (Fox and Weisberg, 2019) and a Pearson correlation test using the R function cor.test (**Table S5**).

#### 2.4.4 Data stratification

To test the performance over an extended range of conditions and cultivars, we stratified the data gathered from the cauliflower and spinach fields according to different levels for calibration. The levels at which data was stratified are:

• All fields together for calibration for spinach or cauliflower (All).
• Splitting the data according to the cultivars (Spinach: Eagle and Whale, Cauliflower: David and Giewont). For spinach, data was split into Eagle and whale, and no other varieties, since for these varieties three or more fields were monitored.
• Splitting the data according to growing period (First: April – July, and Second: July-October).
• Splitting the data according to growing period and cultivars (David 1, David 2, Giewont 1 and Giewont 2).

### 2.5 Output uncertainty

After calibration we tested the model performance by means of calculating uncertainty on model simulation for a selected field during ten different years and for two different soil types (loam and loamy sand). 5000 parameter vectors were randomly selected from the posterior distributions from the Bayesian calibration of all available data (all fields) or subsets from that data (cultivars and growing periods) for each year and soil type. The likeliness of the occurrence of certain yield or biomass output values could therefore be assessed. For spinach, the biomass was calculated at day 38 after sowing, while for cauliflower, yield was calculated at 84 days after planting. Differences between subsetting during calibration could then be visualized in terms of the mean output values and spread (Mean Absolute Deviation, MAD) when subsetting for cultivation period or cultivars. In this study, we determined whether data subsetting according to cultivar and/or cultivation period was superior to keeping all data together to perform calibration for each crop type (spinach and cauliflower).

## 3 Results

### 3.1 Morris method to determine influential parameters

**Figure 2:**
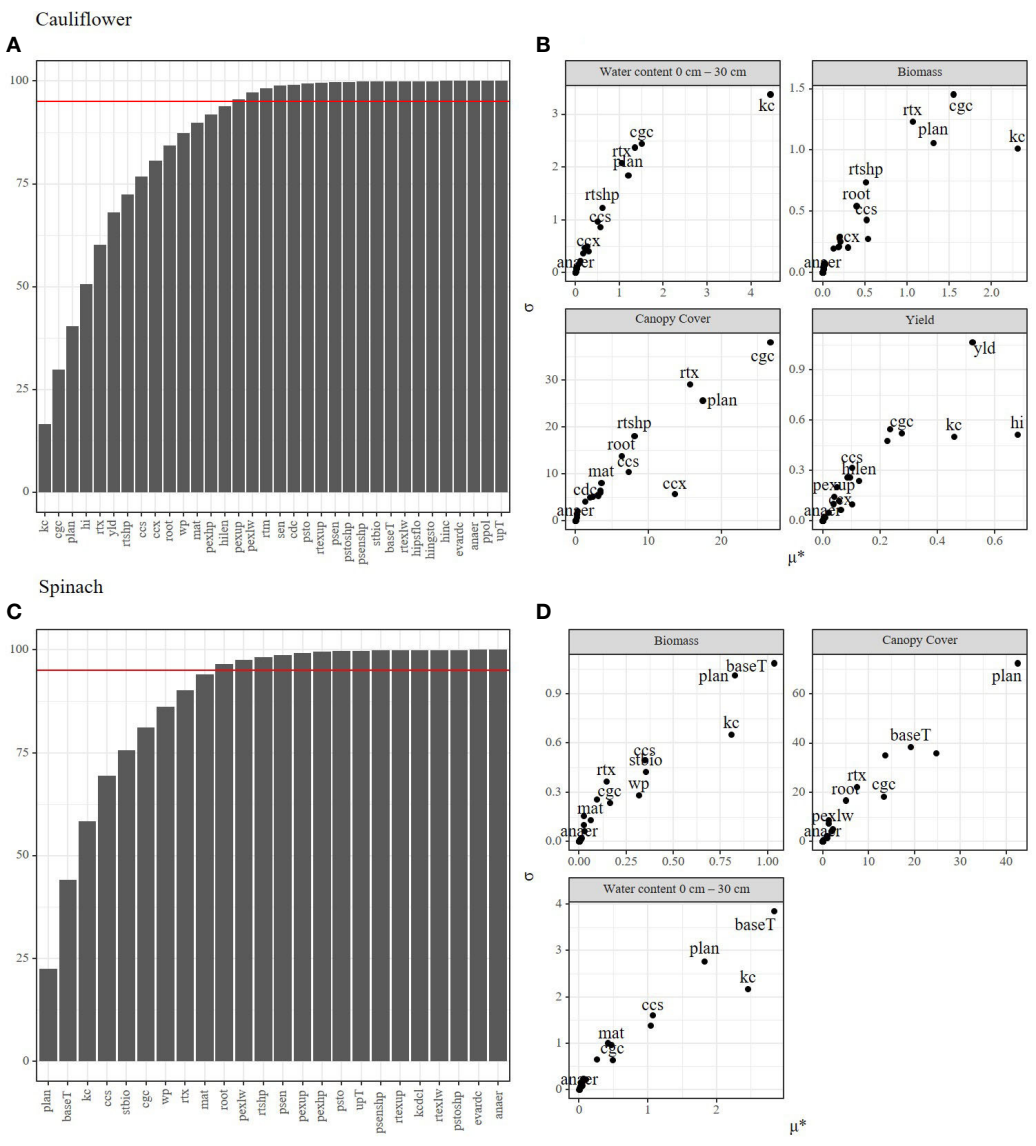
Morris method applied for the spinach and cauliflower fields under study for determining the most influential parameters. Plots **(A, B)** show the cumulative sensitivity of the parameters on the model outputs (**A** for cauliflower, **B** for spinach). The horizontal red line indicates 95 percent of the total sensitivity for determining the most influential parameters to be used in the Bayesian calibration. Plots **(C, D)** show the sensitivity (μ*) calculated for every output variable separately (**C** for cauliflower, **D** for spinach). X-axis indicate the sensitivity of each parameter on the model output, the Y-axis (σ) indicates interaction and non-linear effects of each parameter.

The Morris method produces two important metrics to describe the effects of parameters on the model output: the mean elemental effect (µ*), and the interaction effect (σ). Generally, parameters with a large μ* also display larger interaction or non-linear effects on the model output (**Figures 2C, D**). Multiple parameters (*plan, cgc, kc, ccs, wp*) showed a large impact on all output variables in both spinach and cauliflower, while other parameters (*anaer, evardc, psenshp*) had little or no impact on the model output of both crops. In contrast, some parameters (*baseT, stbio*) had a large impact on spinach while the impact on cauliflower was low. Additionally, since spinach has a short growing period, several parameters (*sen, yld, cdc, hilen, hi, hinc, hingsto, hipsflo*) concerning senescence or yield were not considered or influential for this crop type, while multiple have a large impact on cauliflower (*yld, hi, hilen*). The pooled output variables indicated fifteen parameters constituting more than 95% of the variation in output for cauliflower and only ten parameters for spinach (**Figures 2A, B**). These parameters were further used for the Bayesian calibration.

### 3.2 Bayesian calibration: Posterior distributions

#### 3.2.1 Posterior distributions spinach

**Figure 3:**
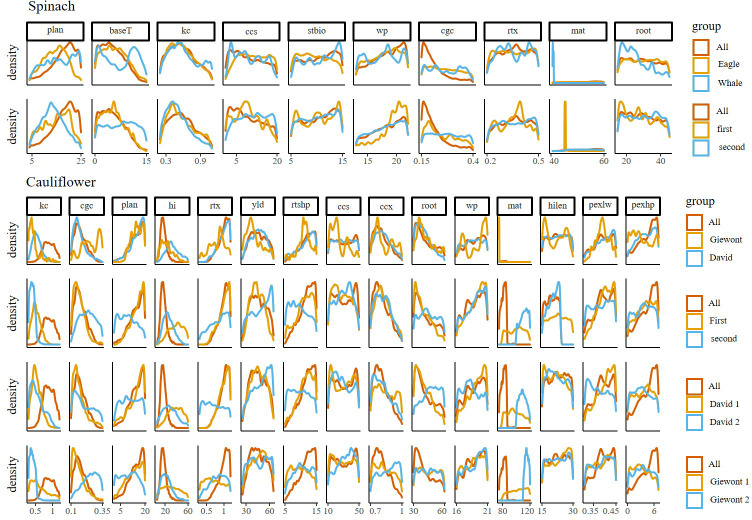
Plots showing the uncertainty of the parameters when splitting data into growing period (First, second), variety (Eagle, Whale, Giewont and David) or both growing period and variety (David 1, David 2, Giewont 1 and Giewont 2) of important parameters of the AquaCrop model for spinach and cauliflower. Posterior distributions calculated by the MCMC Bayesian calibration for spinach and cauliflower for the parameters explaining more than 95 percent of the model sensitivity. Rows indicate the Bayesian calibration determined through the different levels (Period, Year and Cultivar). On every plot a red reference line is plotted where all fields were pooled together for calibration.

The posterior distributions of spinach parameters show that multiple parameters (*root, rtx, stbio, css, wp*) are characterized by a wide spread and the absence of a clear peak, which points towards a large uncertainty on these parameters (**Figure 3**). Like the parameter kc, they also do not display a large difference between calibrations with pooled or subsetted data (**Table S3**), although kc does have a clear maximum in its distribution. In contrast, the parameters *plan, cgc* and *baseT* have clearer differences between the different calibrations, and the calibrations done on the pooled data (‘All’) tend to result in a narrower distribution. Finally, the parameter *mat* has a marked peak for some of the subsets.

#### 3.2.2 Posterior distributions cauliflower

There were clear differences in posterior distribution for multiple parameters (*plan, kc, cgc, hi, mat*) in cauliflower as well (**Figure 3**; **Table S4**). Interestingly, the distribution for plan was different when only data from the second growth period were used for model calibration, regardless of the cultivar, while for *hi*, it was different for the first growth period. Further, the distribution for *kc* was completely different when the pooled data was used, while all other subsets of data resulted in similar distributions. The distributions of the parameter *cgc* was only different for the cultivar Giewont in the second growth period. Multiple parameters also display a wide, almost uniform distribution (*hilen, wp, pexlw, ccs*), with little or no difference between calibrations. For the parameters *pexhp, rtx* and root, the use of all data in the calibration resulted in a narrower distribution, as compared to subsetted data.

### 3.3 Spinach and cauliflower output uncertainty

**Figure 4:**
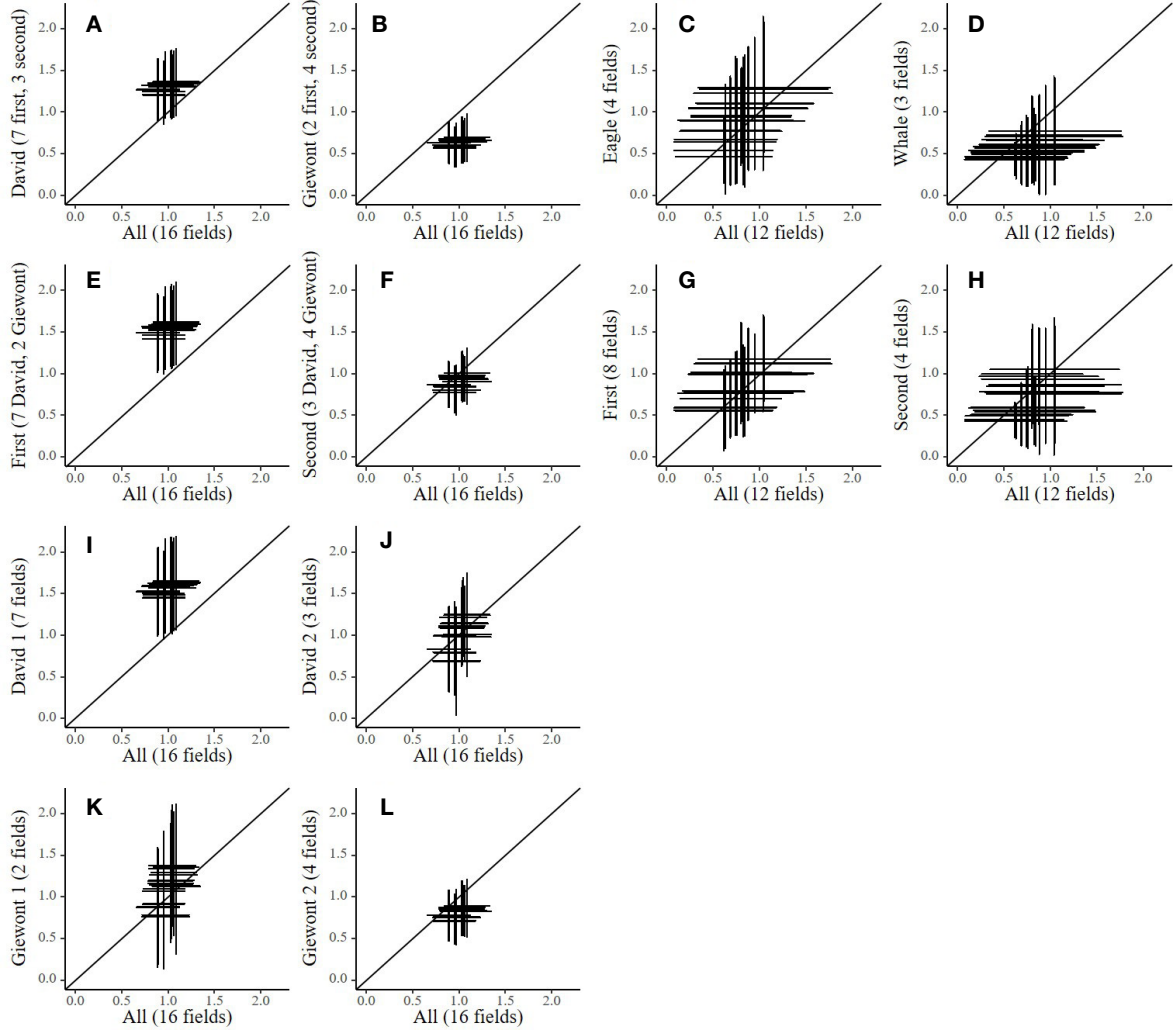
Plots showing the propagated simulation uncertainty (MAD) on yield output (t DM ha^-1^) at day 84 after planting for cauliflower (subplots **A–H**) and day 38 after sowing for spinach (subplots **I–L**) when splitting the calibration data into period and variety plotted as compared to calibrations performed on all available data. Every cross indicates output uncertainty (the mean plus or minus the MAD) calculated based on 5000 random samples from the posterior distributions with calibrations performed with multiple subsets of data for spinach (All, first, second, Eagle and Whale) and cauliflower (All, David, Giewont, first, second, David 1, David 2, Giewont 1 and Giewont 2). Every plot contains twenty crosses for conditions in which simulations were performed (ten years (2009 - 2018) in two different soil types (loam and loamy sand)). The number of fields included in the subset data are indicated in the y axis title.

The uncertainty, represented by the MAD, on the yield of spinach and cauliflower are large and may scale up to more than half a ton ha^-1^ (**Figure 4**). The horizontal lines display the MAD on the simulated output based on the calibration of all available data (All), while the vertical lines are the MAD on the output from the calibration based on the subsets of data. In spinach (**Figures 4I–L**), no substantial differences between the studied levels can be observed, partly because of the large uncertainty. Strikingly, the uncertainty on the output seems at least as large for the calibration based on all available data as for the calibration based on the four subsets of data for spinach.

In contrast, cauliflower generally shows a smaller uncertainty for the calibration based on all available data. However, the mean of these distributions differs from the 1:1 line when splitting the data at cultivar and cultivation period level (cultivar level: David vs Giewont, cultivation period level: first vs second). For example, the calibration based on all data from the cultivar David (**Figure 4A**) result in higher mean outputs for yield, while the calibration based on all data from Giewont (**Figure 4B**) shows lower mean outputs, as compared to the calibration done on all data. Also, the calibrations based on all data from the first growth period (**Figure 4C**), and the calibrations based on de data from David in the first growth period (**Figure 4E**) show a higher mean yield prediction.

## 4 Discussion

### 4.1 Spinach

Splitting the data per cultivar does not contribute to a lower prediction uncertainty spinach (**Figure 4**). This can be explained by the fact that spinach is a fast-growing crop that only produces leaves as biomass. As many relevant parameters in AquaCrop for this early stage of crop development are conservative, these parameters should be fairly stable in different environmental conditions and across different cultivars (Raes et al., 2018). Nevertheless, clear differences in posterior distributions arise for some parameters on all studied levels (*plan, mat*) (**Figure 3** and **Table S3**). And, more importantly, there are large uncertainties in model output. The slight differences in posterior parameter distribution between calibrations (**Figure 3** and **Table S3**) do not result in clear differences between the uncertainties calculated on the model output (**Figure 4**). When performing model simulations with the parameter values with the highest probability based on calibrations with all available data, the model performance seems to be rather low (**Figure S1**). However, while uncertainty on model output is not reduced by including more fields as calibration data, the uncertainty on a number of important parameters (*cgc, baseT*) improved (**Figure 3**).

A substantial part of the large uncertainty in the model simulations can be attributed to the fact that the spinach crop was harvested during the stage of fast growth (38 days after sowing). This inherently results in substantial differences in the observations of biomass. Additionally, interaction effects between parameters may account for a large portion of the uncertainty. For example, the ccs and cgc parameters show large interaction effects (**Figure 2**. This is also visible from their rather uniform distributions without any clear peaks (except the *cgc* parameter shows a clearer peak when all data is pooled together; **Figure 3**). Similarly, the parameters *stbio* and *baseT*, and *root* and *rtx* are interacting parameters. Also because of the large uncertainties on the model output, it is difficult to decide whether calibration is ideally performed at the cultivar level or if data should be pooled together. Potentially, some cultivar specific parameters are ideally estimated per cultivar, and more conservative parameters (which should be stable across cultivars) are better estimated based on all available data.

To break the dependency and interactions between parameters, the availability and quality of the experimental data is crucial. First, the quality of the gathered data may need to be improved since farmers’ fields were monitored in uncontrolled conditions, meaning a larger error on measurements is inherently present. E.g., the uncertainty on the sowing density of spinach (1 190 000 – 2 500 000 plants ha^-1^), causes a difficult assessment of the surface area of the individual seedlings (*ccs*) and canopy growth coefficient (*cgc*).

Second, regarding data availability, parameters may not be identifiable based on the gathered data (Coudron et al., 2021), meaning important moments in development were not be caught during the field measurements. This may be because measurements were not performed frequently enough or because certain events did not occur in the field trials. E.g., drought periods to derive water stress coefficients.

### 4.2 Cauliflower

In contrast with spinach, for cauliflower, we observed clear differences between posterior distributions as a result of data subsetting (**Figure 3** and **Table S4**). Consequently, these differences also resulted in differences in distributions from the simulation outputs, based on data splitted per cultivar or growing period (**Figures 4A–D**). However, further splitting of the data (by cultivar and growth period) results in larger uncertainties on the model output and less clear differences between the data subsets (**Figures 4F, H**). In these cases the number of data points probably becomes too small for proper calibration (Coudron et al., 2021). **Figures 4A, B** suggest that a cultivar dependent calibration is advisable, as both calibrations deviate from each other and from the calibration based on all data. Interestingly, the calibration based on data from the first growth period also deviates from the calibration with all data (**Figure 4C**), whereas the calibration with data from the second growth period does not (**Figure 4D**). However, as the data was collected on farmers’ fields, the experimental set-up was not completely balanced, and data available for the first growth period was dominated by one cultivar (7 fields with David, and only 2 for Giewont). Our results therefore suggest that, if sufficient data is available, calibration is best performed at the cultivar level for cauliflower.

Also in the case of cauliflower, uncertainties may be attributed to errors on field measurements which are performed in non-controlled conditions, interacting parameters which are difficult to determine independently, field heterogeneity, model errors etc. A full uncertainty study should be performed to determine the exact uncertainty sources and how these may be reduced (Wallach et al., 2017). However, errors on model output are smaller than spinach. This may be caused by multiple factors. First, cauliflower is not as fast-growing as spinach and harvest is performed closer to the saturation phase (84 days after planting). Second, the calibration data for spinach contained more cultivars than the calibration data gathered for cauliflower.

Even though uncertainties on model simulations from cauliflower appear not as large as spinach, field specific adjustments may still be needed to capture field specific growth dynamics.

### 4.3 Implications for crop modelling and for farmers: Adjust model parameters with real time data

AquaCrop is a simulation model that is commonly used in decision support tools (Cusicanqui et al., 2013). Our study has demonstrated that uncertainty on the model simulations can be quite large, given that parameter estimation was done on observational data from farmers’ fields (**Figure 4**). This highlights the need for good calibration practices such that end-users can determine if the simulations are sufficiently reliable for their particular applications (Seidel et al., 2018; Gao et al., 2020), and model misspecification can be avoided (Wallach, 2011). Because of the large model uncertainties, multiple additional sources of information are recommended to adjust and improve model simulations for farmers’ fields based on real time observations (Zhang et al., 2021). This should capture field, cultivar and growing period specific dynamics which cannot always be captured based on collected calibration data. This includes satellite images, biogeochemical and texture analysis of soil samples, regular canopy cover pictures (e.g. taken by the farmer) (Dehnen-Schmutz et al., 2016) etc. For example, satellite images may give us a better understanding of the canopy development in fairly high resolution and can therefore be useful to estimate multiple developmental parameters with more accuracy. Indeed, Sentinel satellite images can be used to monitor the crop performance on a resolution of 10m by 10m (Vannoppen and Gobin, 2022) and can be used to adjust AquaCrop model simulations (Sallah et al., 2019). From the posterior distributions, it is clear that the parameters *ccs* and *cgc* are difficult to estimate well, based on the collected calibration data. Such parameters strongly depend on the management practices by the farmer. In case of AquaCrop, these parameters can be better determined with field specific canopy cover measurements. Therefore, remotely sensed data such as satellite and/or drone images and extra ground observations by farmers, scientists, consultants or other stakeholders therefore play an important role in reducing the uncertainty on these parameters and improving model performance in near-real time. For example, Sentinel 2 satellite images are freely available from Terrascope (https://terrascope.be/) and have been used for monitoring potato growth and canopy cover (Vannoppen and Gobin, 2022). Therefore, being a valuable source of information for calibration of models such as AquaCrop. Additionally, local soil texture analysis or information about the ground water depth may prove to be important sources of information to reduce errors in the AquaCrop model prediction for a specific field (Vanuytrecht et al., 2014; Zhao et al., 2020).

Multiple platforms exist which bring these data sources together and which aim at aiding the farmer in the monitoring of their fields (Minet et al., 2017). These platforms are ideal to implement crop models such as AquaCrop that can make use of all the data sources to perform more accurate simulations with real time data. This not only reduces uncertainty on model simulations, but also allows the option for more general calibrations on crop varieties which can be fine-tuned with the available data.

## 5 Conclusion

We tested the need for cultivar and growth period specific calibration of the AquaCrop model for cauliflower and spinach with a Bayesian calibration approach in Belgian climatic conditions. Our results indicate a need for cultivar specific calibration for cauliflower, whereas for spinach, splitting the data per cultivar or pooling the data together did not improve uncertainty on the model simulations. However, due to uncertainties arising from field specific soil/weather conditions, measurement errors on calibration data, and constraints associated with data collection on farmers’ fields, it is advised to make real time field specific adjustments to simulations when using AquaCrop in a decision support tool which captures field, growing period and cultivar specific dynamics. Remotely sensed or ground data may be invaluable information to reduce uncertainty on model simulations.

## Data availability statement

The datasets presented in this study can be found in online repositories. The names of the repository/repositories and accession number(s) can be found below: https://zenodo.org/record/7307969#.Y2usRHbMIuU.

## Author contributions

WC: Conceptualization, data collection, Writing- Original draft preparation. TD, KV and PD: Conceptualization, Writing–Review & Editing, Supervision. SP, AG and CB: Writing–Review & Editing. TD: Data Collection, Writing–Review & Editing. All authors contributed to the article and approved the submitted version.
