# Supplementary material for: Usefulness of cultivar-level calibration of AquaCrop for vegetables depends on the crop and data availability
Source: Front Plant Sci. 2023 Mar 9;14:1094677. doi: 10.3389/fpls.2023.1094677 (PMC10034377; doi:10.3389/fpls.2023.1094677)
Supplement: Supplementary file 1 [file DataSheet_1.docx]

Supplementary Material

Table S1: Fields monitored in Flanders for the calibration of the AquaCrop model for spinach and cauliflower. Fields with ID 10 and 11 were chosen for the calculation of the output uncertainty (section 2.5).

|  |  |  |  |  |  |  |  |  | **Number of measurements** | | | |
| --- | --- | --- | --- | --- | --- | --- | --- | --- | --- | --- | --- | --- |
| **ID** | **LAT** | **LON** | **Crop** | **Cultivar** | **Plant date** | **Harvest date** | **density** | **Soil type** | **Yield** | **Biomass** | **CC** | **WC 0-30** |
| 11 | 50.811 | 2.807 | Cauliflower | David | 6/04/2019 | 28/06/2019 | 27487 | Loam | 2 | 2 | 5 | 6 |
| 12 | 50.968 | 3.476 | Cauliflower | David | 28/04/2019 | 7/07/2019 | 27600 | Sandyloam | 2 | 3 | 5 | 5 |
| 13 | 50.936 | 2.851 | Cauliflower | David | 17/04/2019 | 1/07/2019 | 25950 | Loam | 3 | 3 | 6 | 6 |
| 14 | 50.978 | 2.899 | Cauliflower | David | 16/07/2019 | 22/10/2019 | 26592 | Loam | 2 | 3 | 7 | 8 |
| 15 | 50.943 | 3.406 | Cauliflower | David | 27/07/2019 | 22/10/2019 | 31909 | Sandyloam | 2 | 4 | 6 | 8 |
| 16 | 51.02 | 3.462 | Cauliflower | David | 11/07/2019 | 5/10/2019 | 32500 | Loam | 1 | 6 | 6 | 6 |
| 17 | 51.017 | 3.059 | Cauliflower | David | 17/07/2019 | 11/10/2019 | 26365 | Clay | 3 | 5 | 8 | 8 |
| 18 | 51.018 | 2.916 | Cauliflower | David | 15/04/2019 | 1/07/2019 | 28292 | Loamysand | 3 | 3 | 5 | 6 |
| 27 | 50.857 | 3.426 | Cauliflower | David | 29/04/2020 | 7/07/2020 | 42000 | Sand | 2 | 4 | 6 | 6 |
| 28 | 50.98 | 3.039 | Cauliflower | David | 19/04/2020 | 24/06/2020 | 24885 | Sandyloam | 4 | 4 | 6 | 7 |
| 29 | 50.986 | 3.059 | Cauliflower | Giewont | 7/07/2020 | 19/10/2020 | 25107 | Loam | 3 | 6 | 9 | 9 |
| 33 | 51.015 | 3.18 | Cauliflower | Giewont | 22/04/2021 | 7/07/2021 | 21805 | Loam | 2 | 3 | 7 | 7 |
| 34 | 50.991 | 3.16 | Cauliflower | Giewont | 24/06/2021 | 27/09/2021 | 24353 | Loam | 3 | 4 | 7 | 6 |
| 47 | 50.97 | 3.475 | Cauliflower | Giewont | 27/04/2021 | 16/07/2021 | 28542 | Loamysand | 1 | 2 | 5 | 5 |
| 48 | 50.884 | 3.382 | Cauliflower | Giewont | 13/07/2020 | 25/10/2020 | 28542 | Loamysand | 3 | 5 | 7 | 8 |
| 49 | 50.863 | 3.375 | Cauliflower | Giewont | 20/07/2021 | 28/10/2021 | 28542 | Sandyloam | 3 | 2 | 6 | 7 |
| 7 | 50.867 | 3.389 | Spinach | Berkner | 16/04/2019 | 3/06/2019 | 2172500 | Loamysand |  | 2 | 3 | 4 |
| 8 | 51.022 | 3.52 | Spinach | Spirico | 2/04/2019 | 27/05/2019 | 1995000 | Sandyloam |  | 4 | 4 | 4 |
| 9 | 50.945 | 3.422 | Spinach | Puma | 13/05/2019 | 22/06/2019 | 2020000 | Sandyloam |  | 3 | 5 | 5 |
| 10 | 51.038 | 3.07 | Spinach | Whale | 1/04/2019 | 27/05/2019 | 1847500 | Sandyloam |  | 3 | 3 | 5 |
| 23 | 50.867 | 3.389 | Spinach | Whale | 15/04/2020 | 28/05/2020 | 1897727 | Loam |  | 2 | 5 | 4 |
| 24 | 50.953 | 3.215 | Spinach | Eagle | 8/04/2020 | 20/05/2020 | 2520000 | Sandyloam |  | 4 | 11 | 7 |
| 25 | 50.938 | 3.178 | Spinach | Eagle | 22/07/2020 | 1/09/2020 | 1190000 | Loamysand |  | 4 | 5 | 6 |
| 26 | 50.881 | 3.266 | Spinach | Bonobo | 30/07/2020 | 14/09/2020 | 1750000 | Loam |  | 5 | 6 | 7 |
| 32 | 51.013 | 3.178 | Spinach | Whale | 14/04/2021 | 4/06/2021 | 2500000 | Sandyloam |  | 5 | 7 | 9 |
| 46 | 50.958 | 3.476 | Spinach | Sacramento | 31/07/2020 | 14/09/2020 | 1281250 | Loam |  | 2 | 3 | 4 |
| 51 | 50.974 | 3.301 | Spinach | Eagle | 11/04/2021 | 4/06/2021 | 2135417 | Sandyloam |  | 2 | 5 | 3 |
| 52 | 51.015 | 3.059 | Spinach | Eagle | 14/08/2021 | 20/09/2021 | 1221250 | Sandyloam |  | 3 | 5 | 6 |

*Table S2: Parameter ranges used for sensitivity analysis and Bayesian calibration for spinach and cauliflower. Distributions for calibration were assumed uniform between the lower and upper boundaries.*

| **Crop parameters** | **Parameter ranges Spinach** | **Parameter ranges cauliflower** | **Units** | **Parameter description** |
| --- | --- | --- | --- | --- |
| **Development** | |  |  |  |
| *mat* | 45-65 | 90-120 | Days (d) | Total length of crop cycle from sowing or planting to maturity in calendar days |
| *plan* | 4-25 | 1-20 | d | Period from sowing to emergence or from planting to recovered transplant |
| *ccs* | 1-20 | 10-50 | cm^2^ | Soil surface covered by an individual at planting or 90 % emergence |
| *cgc* | 0.15-0.4 | 0.1-0.35 | Fraction d^-1^ | Increase in canopy cover |
| *ccx* | 1 | 0.7-1 | Fraction of 1 | Maximum canopy cover |
| *sen* | - | 70-100 | Days | Period from planting to start senescence |
| *yld* | - | 25-65 | Days | Period from planting to start of yield formation |
| *cdc* | - | 0.05-0.18 | Fraction d^-1^ | Decrease in canopy cover fraction per day |
| *hilen* | - | 15-30 | d | Period of harvest index building-up during yield formation |
| *root* | 15-45 | 30-60 | d | Development root period from sowing to maximum rooting depth |
| *rtm* | 0.1 | 0.1-0.2 | m | Minimum effective rooting depth |
| *rtx* | 0.2-0.5 | 0.3-1.20 | m | Maximum effective rooting depth |
| *rtshp* | 10-15 | 5-15 | - | Shape factor describing root zone expansion |
| *rtexup* | 0.02-0.08 | 0.02-0.08 | m^3^ m^-3^ soil d^-1^ | Maximum root water extraction in top quarter of root zone |
| *rtexlw* | 0.015-0.06 | 0.015-0.06 | m^3^ m^-3^ soil d^-1^ | Maximum root water extraction in bottom quarter of root zone |
| *baseT* | 0-15 | 1-3 | °C | Base temperature below which crop development does not progress |
| *upT* | 24-29 | 24-28 | °C | Upper temperature above which crop development no longer increases with an increase in temperature |
| **Transpiration** |  |  |  |  |
| *kc* | 0.2-1.1 | 0.3-1.25 | - | Crop coefficient when canopy is complete but prior to senescence |
| *kcdcl* | 0.075-0.225 | 0.075-0.225 | % d^-1^ | Decline of crop coefficient as a result of ageing, and nitrogen deficiency |
| *evardc* | 40-80 | 40-80 | - | Effect of canopy cover in reducing soil evaporation in late season stage |
| **Production** |  |  |  |  |
| *wp* | 13-22 | 16-21 | g m^-2^ | Water productivity normalized for ETo and CO_2_ |
| *hi* | - | 5-60 | % | Reference harvest index (HI) |
| **Stress** |  |  |  |  |
| *pexup* | 0.05-0.3 | 0.01-0.3 | Fraction TAW | Upper threshold of soil water depletion limiting canopy expansion |
| *pexlw* | 0.45-0.7 | 0.3-0.45 | Fraction TAW | Lower threshold of soil water depletion limiting canopy expansion |
| *pexshp* | 1.5-4.5 | 0-5 | - | Shape factor for water stress limiting canopy expansion (0.0 = straight line) |
| *psto* | 0.5-0.65 | 0.4-0.6 | Fraction TAW | Upper threshold of soil water depletion limiting stomatal conductance |
| *pstoshp* | 1.5-4.5 | 0-5 | - | Shape factor for water stress limiting stomatal conductance (0.0 = straight line) |
| *psen* | 0.7-0.85 | 0.65-0.8 | Fraction TAW | Upper threshold of soil water depletion inducing early canopy senescence |
| *psenshp* | 1.5-4.5 | 0-5 | - | Shape factor for water stress inducing early canopy senescence (0.0 = straight line) |
| *anaer* | 4-8 | 4-8 | Vol% | Anaerobic point below saturation limiting aeration |
| *hipsflo* | - | 1-3 | % | Possible increase of harvest index due to water stress before flowering |
| *hingsto* | - | 5-15 | - | Coefficient for negative impact of stomatal closure during yield formation on HI |
| *hinc* | - | 2-8 | % | Allowable maximum increase of HI |
| *stbio* | 5-15 | 5-9 | GDD d^-1^ | Minimum growing degrees for full biomass production |


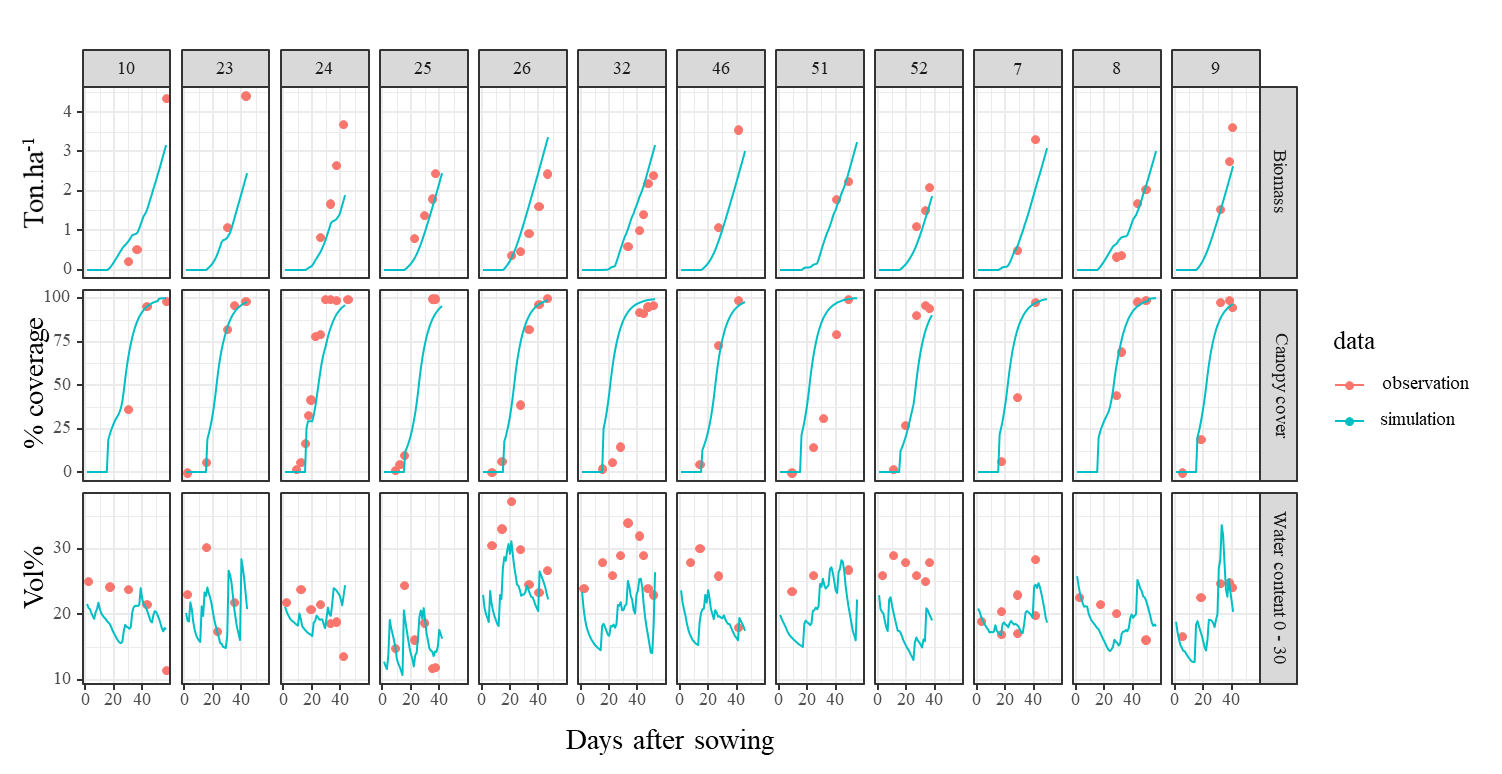


*Figure S1: Comparison between observed values and simulated values. Simulations were performed with the best parameterset based on calibrations performed with all available data of spinach. The following goodness-of-fit metrics for the biomass and canopy cover output were performed for these simulations: Mean absolute error (MAE), Root Mean Squared Error (RMSE), coefficient of determination (R^2^) and model efficiency (EF). Biomass: MAE = 0.6009, RMSE = 0.7604, R^2^ = 0.5831, EF = 0.1075. Canopy cover: MAE = 12.21, RMSE = 19.78, R^2^ = 0.7718, EF = 0.7456*

*Table S3: D-statistics of the Kolmogorov-Smirnov test comparing the distance between posterior distributions of parameters between calibrations performed with different data groups for spinach. Colors indicate the similarity between the distributions, green means more similar, while red means less similar.*

| **Groups** | | **plan** | **baseT** | **kc** | **ccs** | **stbio** | **wp** | **cgc** | **rtx** | **mat** | **root** |
| --- | --- | --- | --- | --- | --- | --- | --- | --- | --- | --- | --- |
| **All** | **First** | 0.232 | 0.051 | 0.059 | 0.073 | 0.042 | 0.217 | 0.251 | 0.068 | 0.911 | 0.059 |
| **All** | **Second** | 0.391 | 0.266 | 0.149 | 0.135 | 0.033 | 0.027 | 0.382 | 0.028 | 0.093 | 0.044 |
| **All** | **Eagle** | 0.303 | 0.087 | 0.017 | 0.147 | 0.119 | 0.067 | 0.340 | 0.050 | 0.249 | 0.044 |
| **All** | **Whale** | 0.192 | 0.312 | 0.069 | 0.049 | 0.064 | 0.108 | 0.344 | 0.057 | 1.000 | 0.206 |
| **first** | **Second** | 0.171 | 0.263 | 0.105 | 0.138 | 0.036 | 0.231 | 0.145 | 0.093 | 0.948 | 0.088 |
| **Eagle** | **Whale** | 0.221 | 0.268 | 0.078 | 0.125 | 0.164 | 0.068 | 0.073 | 0.049 | 0.974 | 0.231 |

*Table S4: D-statistics of the Kolmogorov-smirnov test comparing the distance between posterior distributions of parameters between calibrations performed with different data groups for cauliflower. Colors indicate the similarity between the distributions, green means more similar, while red means less similar.*

| **Groups** | | **kc** | **cgc** | **plan** | **hi** | **rtx** | **yld** | **rtshp** | **ccs** | **ccx** | **root** | **wp** | **mat** | **hilen** | **pexlw** | **pexhp** |
| --- | --- | --- | --- | --- | --- | --- | --- | --- | --- | --- | --- | --- | --- | --- | --- | --- |
| **All** | **David** | 0.754 | 0.104 | 0.074 | 0.686 | 0.059 | 0.045 | 0.061 | 0.038 | 0.098 | 0.109 | 0.057 | 0.749 | 0.027 | 0.064 | 0.123 |
| **All** | **Giewont** | 0.810 | 0.496 | 0.088 | 0.338 | 0.409 | 0.236 | 0.233 | 0.270 | 0.166 | 0.107 | 0.189 | 0.940 | 0.140 | 0.107 | 0.285 |
| **All** | **First** | 0.781 | 0.096 | 0.075 | 0.768 | 0.037 | 0.098 | 0.123 | 0.097 | 0.101 | 0.094 | 0.098 | 0.821 | 0.391 | 0.092 | 0.143 |
| **All** | **Second** | 0.954 | 0.383 | 0.407 | 0.479 | 0.310 | 0.156 | 0.361 | 0.084 | 0.110 | 0.188 | 0.058 | 1.000 | 0.104 | 0.124 | 0.195 |
| **All** | **David 1** | 0.753 | 0.126 | 0.054 | 0.758 | 0.043 | 0.030 | 0.074 | 0.050 | 0.191 | 0.082 | 0.108 | 0.801 | 0.073 | 0.109 | 0.206 |
| **All** | **David 2** | 0.652 | 0.227 | 0.301 | 0.510 | 0.489 | 0.162 | 0.307 | 0.079 | 0.074 | 0.281 | 0.147 | 1.000 | 0.064 | 0.204 | 0.253 |
| **All** | **Giewont 1** | 0.561 | 0.171 | 0.130 | 0.533 | 0.500 | 0.092 | 0.319 | 0.046 | 0.157 | 0.172 | 0.052 | 0.873 | 0.064 | 0.108 | 0.273 |
| **All** | **Giewont 2** | 0.960 | 0.514 | 0.342 | 0.316 | 0.367 | 0.078 | 0.239 | 0.099 | 0.305 | 0.170 | 0.039 | 1.000 | 0.024 | 0.107 | 0.224 |
| **David** | **Giewont** | 0.393 | 0.550 | 0.107 | 0.398 | 0.359 | 0.272 | 0.210 | 0.285 | 0.123 | 0.098 | 0.234 | 0.968 | 0.119 | 0.163 | 0.210 |
| **First** | **Second** | 0.431 | 0.457 | 0.455 | 0.457 | 0.275 | 0.250 | 0.262 | 0.088 | 0.036 | 0.241 | 0.047 | 0.477 | 0.389 | 0.180 | 0.072 |
| **David 1** | **David 2** | 0.115 | 0.252 | 0.294 | 0.296 | 0.489 | 0.161 | 0.263 | 0.069 | 0.129 | 0.317 | 0.245 | 0.579 | 0.043 | 0.246 | 0.075 |
| **Giewont 1** | **Giewont 2** | 0.501 | 0.582 | 0.216 | 0.319 | 0.190 | 0.071 | 0.104 | 0.115 | 0.153 | 0.027 | 0.070 | 0.693 | 0.069 | 0.017 | 0.076 |

*Table S5: The homogeneity of variance (Levene test) (Liu et al. 2018) and Pearson correlation of the residues for a representative field for every studied cultivar to determine whether errors for different response variables have the same distribution and are correlated (Wallach et al. 2021). These tests were performed for a representative field for the variables: canopy cover (CC), Biomass, and water content in the 0 – 30 soil layer. Errors were calculated as the difference between observations and simulations performed with the most likely parameterset calibrated based on all available data for each crop.*

| **ID** | **Cultivar** | **Output variables** | **Pearson correlation** | **Pearson correlation p-value** | **Levenetest p-value** |
| --- | --- | --- | --- | --- | --- |
| 32 | Whale | CC - Biomass | 0.531 | 0.47 | 0.08618 |
|  |  | CC - Water content | -0.653 | 0.35 |  |
|  |  | Water content - Biomass | 0.095 | 0.90 |  |
| 52 | Eagle | CC - Biomass | 0.251 | 0.83 | 0.1933 |
|  |  | CC - Water content | 0.874 | 0.32 |  |
|  |  | Water content - Biomass | -0.25 | 0.84 |  |
| 15 | David | CC - Biomass | -0.992 | 0.008 | 0.01054 |
|  |  | CC - Water content | 0.823 | 0.18 |  |
|  |  | Water content - Biomass | -0.814 | 0.19 |  |
| 29 | Giewont | CC - Biomass | -0.575 | 0.42 | 0.1642 |
|  |  | CC - Water content | -0.816 | 0.18 |  |
|  |  | Water content - Biomass | 0.555 | 0.44 |  |
